# Role of ^99m^Tc-(V)DMSA in Detecting Tumor Cell Proliferation

**Published:** 2007-12-13

**Authors:** Fatma Al-Saeedi

**Affiliations:** Department of Nuclear Medicine, Faculty of Medicine, Kuwait University, Kuwait.

**Keywords:** ^99m^Tc-(V)DMSA, cancer, cell proliferation

## Abstract

Pentavalent technetium-99m dimercaptosuccinic acid (^99m^Tc-(V)DMSA) is a tumor-seeking agent which was introduced to evaluate, image, and manage many types of cancers. In this review, the beginning of, and the most recent applications of using this agent was appraised. The relation with tumor cell detection and proliferation was reported and several mechanisms of uptake of ^99m^Tc-(V)DMSA in tumor cells are described.

## Introduction

Recently attempts have been made to find a more accurate and specific radioactive tracer to image the proliferative activity of various types of cancers, including breast cancer. ^18^F-flurodeoxy glucose (^18^F-FDG) positron emission tomography (PET), ^18^F-flurothymidine (^18^F-FLT), and deoxyuridine has shown unclear results (Rasey et al. 2002; Toyohara et al. 2002; Buck et al. 2002). Pentavalent technetium-99m dimercaptosuccinic acid (^99m^Tc-(V)DMSA) is a new tumor-seeking agent, which was introduced to evaluate, image, and manage medullary carcinoma of the thyroid (Ohta et al. 1984; Clarke et al. 1988). ^99m^Tc-(V)DMSA uptake showed its presence and efficacy in detecting many other types of cancers; such as head and neck, in particular squamous cell carcinoma, and soft tissue tumors (Ohta et al. 1988; Watkinson et al. 1989), breast (Papantoniou et al. 2002; Kashyap et al. 1992), brain (Hirano et al. 1997), lung (Hirano et al. 1995; Atasever et al. 1997), bone (Lam et al. 1997), and in particular, for metastasis and high-grade tumors (Kiratli et al. 1998). However in patients with carcinomas of the liver and gastrointestinal tract, malignant melanoma and lymphoma, the role of ^99m^Tc-(V)DMSA was absent (Ohta et al. 1988). Physiological uptake has been evidenced in the kidneys, nasal mucosa, lacrimal glands and blood pool such as in the heart and vessels. Uptake of ^99m^Tc-(V)DMSA was also reported as normal distribution in the pituitary gland (Watkinson et al. 1990). It’s uptake in the breasts has often been noted. Nakamoto et al. (1997) reported that female breasts should be included in the normal biodistribution of ^99m^Tc-(V)DMSA and should not be considered as pathological.

^99m^Tc-(V)DMSA has a very high affinity for various tumors and increased uptake in tumors, but the mechanism of ^99m^Tc-(V)DMSA uptake in tumors is still not fully elucidated. A working hypothesis based on the metal-complex equilibrium has anticipated the pH sensitive character of ^99m^Tc-(V)DMSA. This character was suggested to be one of the factors that affected its accumulation in cancer cells (Horiuchi et al. 1998a).

Factually, tumours are well known to be more acidic than normal cells (Tannock and Rotin, 1989), *in vivo* ^99m^Tc-(V)DMSA uptake was correlated to the lowering of pH of tumors by glucose administration using Ehrlich ascites tumor cells (EATC)-bearing mice (Horiuchi et al. 1998b). The study found evidence of correlation between the ^99m^Tc-(V)DMSA uptake, which reflects the tumor acidosis, and the ^14^C-deoxyglucose (^14^C-DG) uptake, which reflects the tumor glycolysis and viability in EATC-bearing mice. Horiuchi et al. (1998b) reported that ^99m^Tc-(V)DMSA uptake by tumors was related to glucose-mediated acidosis, and this may reflect tumor cell viability. However, this was only reported by one study and in one type of cell lines (EATC) *in vivo*. This EATC also belongs to well-differentiated cells with very rapid growth rate and capacity of high aerobic glycolysis (Bustamante et al. 1981), and therefore it can not reflect the accurate mechanism of ^99m^Tc-(V)DMSA uptake by tumors.

One study suggested that ^99m^Tc-(V)DMSA uptake by breast tumors is related to proliferative activity in breast, which is directly related to tumor grade (Papantoniou et al. 1999). In addition, Papantoniou et al. (2001) reported that ^99m^Tc-(V)DMSA uptake by breast tumors and several breast lesions is related to the mitotic activity and the cellular proliferation of breast tumors or lesions. Recently a strong correlation between ^99m^Tc-(V)DMSA uptake and cellular proliferation, as measured by Ki-67 expression (Ki-67, a monoclonal antibody that recognizes a labile epitope on a nuclear antigen expressed in cycling cells but not quiescent cells (Sawhney and Hall, 1992) was demonstrated (Papantoniou et al. 2004; Papantoniou et al. 2005). Papantoniou et al. (2004) showed that the proliferative activity is a major independent factor affecting ^99m^Tc-(V)DMSA uptake in breast cancer as determined by Ki-67 expression and suggested that ^99m^Tc-(V)DMSA uptake can be of clinical significance as an *in vivo* indicator of cell proliferation.

One *in vitro* study showed that ^99m^Tc-(V)DMSA uptake in cancer cell lines (human breast cancer; MCF-7, human glioblastoma multiform; G152, human fibrosarcoma; HT1080, lung adenocarcinoma; A549, human amelanomic melanoma; M3DAU, and grade III human glioblastoma; U-87-MG) is closely related to proliferation rate and focal adhesion kinase (FAK). Because proliferation rate and FAK are linked to cancer progression, Denoyer et al. (2005) assumed that *in vivo* ^99m^Tc-(V)DMSA uptake reflects tumor aggressiveness and that ^99m^Tc-(V)DMSA uptake could provide clinicians with preoperative information not always obtainable by mammography.

In addition, its mechanism of uptake is thought to be due to the structural similarity between ^99m^Tc-(V)DMSA core (phosphate-like ion ${\text{TcO}}_{4}^{-3}$) and phosphate ( ${\text{PO}}_{4}^{-3}$) anion as taken by some cancer cells (Yokoyama and Saji, 1980; Wulfrank et al. 1989). ^99m^Tc-(V)DMSA was originally designed as a metabolic mimic of phosphate, able to localize in cancer cells by supposed hydrolysis of the pentavalent DMSA complex within cancer cells to produce the phosphate-like ion ${\text{TcO}}_{4}^{-3}$ (Horiuchi et al. 1986; Yokoyama and Saji, 1980; Yokoyama et al. 1981; Yokoyama et al. 1985), then the hydrolysis of ${\text{TcO}}_{4}^{-3}$ and the subsequent metabolism or whatever mechanism starts in the cancer cells. The possible involvement of phosphate anion in ^99m^Tc-(V)DMSA uptake by tumors was confirmed by the inhibition of the uptake of ^99m^Tc-(V)DMSA in the presence of phosphate ion (Lam et al. 1996).

Phosphate ion carries a negative charge, and so it is weakly accumulated in the cytosol by simple diffusion. However, it enters cells via three different types of NaPi co-transporters: type I NaPi co-transporters or NPT1 in humans, expressed in liver and kidney (Ghishan et al. 1993; Li and Xie, 1995), type II NaPi co-transporters (NPT2 in humans), expressed in brain (Hisano et al. 1997), osteoclasts (Gupta et al. 1996), lung (Magagnin et al. 1993), small intestine and proximal tubes (Hilfiker et al. 1998), and type III NaPi co-transporters, which are a family of cell surface receptors for the Gibbon ape leukaemia virus (GALV) (Kavanaugh and Kabat, 1996). These receptors are highly related to phosphate cotransporters (PiT1 in humans). This protein family is found in several organs and many tumor-forming tissues (Kavanaugh and Kabat, 1996; Kavanaugh et al. 1994; Johann et al. 1992; Miller and Miller, 1994; Uckert et al. 1998).

One study by Palmedo et al. (1996; 2000) demonstrated that ^99m^Tc-(V)DMSA is a marker of phosphate transport and ^99m^Tc-(V)DMSA enters a cancer cell line model specifically via type III NaPi co-transporters (PiT1) (Denoyer et al. 2004). This was recently demonstrated by Denoyer et al. (2004), by comparing ^99m^Tc-(V)DMSA and phosphate uptake kinetics in three cancer cell lines (human breast cancer; MCF-7, human glioblastoma multiform; G152, and human osteosarcoma; MG-63) and showed that ^99m^Tc-(V)DMSA uptake is specifically mediated by PiT1 in cancer cells. From this study, ^99m^Tc-(V)DMSA uptake can be assumed to be cell specific, because MCF-7 and G152 cancer cell lines exhibited the same ^99m^Tc-(V)DMSA uptake, whereas MG-63 cancer cell line showed the highest phosphate accumulation and the lowest ^99m^Tc-(V)DMSA uptake.

## Conclusion

In this review, ^99m^Tc-(V)DMSA capability to detect cancer non invasively was reported. Several studies proposed the mechanism of ^99m^Tc-(V)DMSA uptake that might be involved by cancer cells. In addition, some studies reported its relation to cancer cell proliferation and this uptake might be used as a proliferation marker in cancer cells. However, until now, no strict mechanism has been fully clarified.
